# AtPHT4;4 is a chloroplast-localized ascorbate transporter in *Arabidopsis*

**DOI:** 10.1038/ncomms6928

**Published:** 2015-01-05

**Authors:** Takaaki Miyaji, Takashi Kuromori, Yu Takeuchi, Naoki Yamaji, Kengo Yokosho, Atsushi Shimazawa, Eriko Sugimoto, Hiroshi Omote, Jian Feng Ma, Kazuo Shinozaki, Yoshinori Moriyama

**Affiliations:** 1 Advanced Science Research Center, Okayama University, Okayama 700-8530, Japan; 2 Gene Discovery Research Group, RIKEN Center for Sustainable Resource Science, Yokohama 230-0045, Japan; 3 Department of Membrane Biochemistry, Okayama University Graduate School of Medicine, Dentistry and Pharmaceutical Sciences, Okayama 700-8530, Japan; 4 Institute of Plant Science and Resources, Okayama University, Kurashiki 710-0046, Japan

## Abstract

Ascorbate is an antioxidant and coenzyme for various metabolic reactions *in vivo*. In plant chloroplasts, high ascorbate levels are required to overcome photoinhibition caused by strong light. However, ascorbate is synthesized in the mitochondria and the molecular mechanisms underlying ascorbate transport into chloroplasts are unknown. Here we show that AtPHT4;4, a member of the phosphate transporter 4 family of *Arabidopsis thaliana*, functions as an ascorbate transporter. *In vitro* analysis shows that proteoliposomes containing the purified AtPHT4;4 protein exhibit membrane potential- and Cl^−^-dependent ascorbate uptake. The AtPHT4;4 protein is abundantly expressed in the chloroplast envelope membrane. Knockout of *AtPHT4;4* results in decreased levels of the reduced form of ascorbate in the leaves and the heat dissipation process of excessive energy during photosynthesis is compromised. Taken together, these observations indicate that the AtPHT4;4 protein is an ascorbate transporter at the chloroplast envelope membrane, which may be required for tolerance to strong light stress.

## Abstract (web summary)

In plants, ascorbate is synthesized in the mitochondria yet plays essential roles as an antioxidant in the chloroplast. Here, Miyaji *et al.* show that AtPHT4;4 is a chloroplast envelope ascorbate transporter and suggest it is required for dissipation of excess energy under light stress.

## Graphical abstract

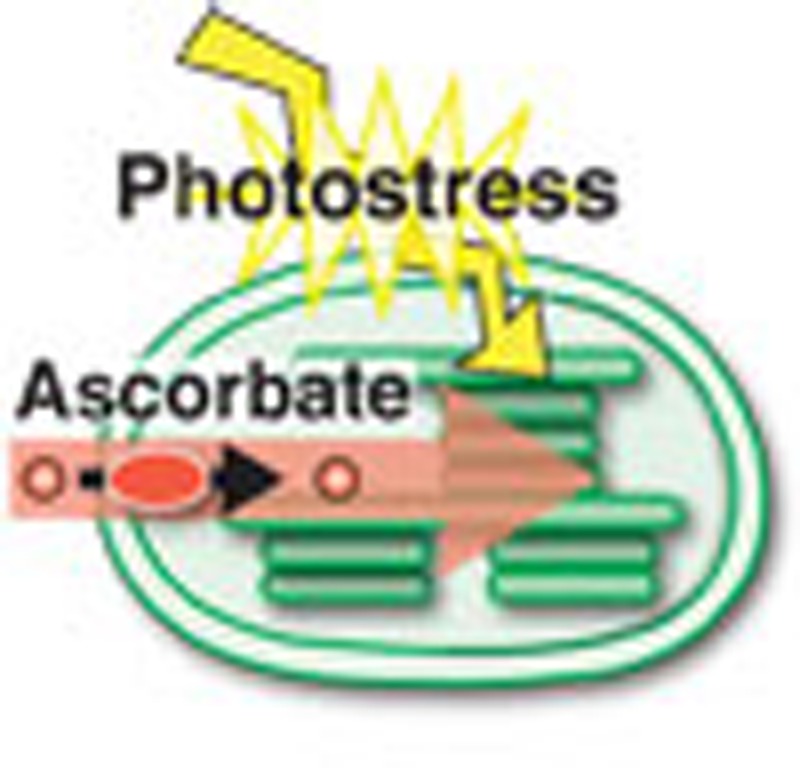

Ascorbate (vitamin C) is an antioxidant and coenzyme for a number of metabolic reactions in living organisms^1^,^2^. Primates, including humans, have a defect in the enzyme responsible for ascorbate synthesis, L-gulono-1,4-lactone oxidase, and must therefore acquire ascorbate via the diet to maintain homeostasis. In plants, however, ascorbate is synthesized in the mitochondria in response to external stresses, distributed throughout the cells, and confers stress tolerance^2^,^3^,^4^. In particular, chloroplasts contain high concentrations of ascorbate (10–50 mM)^4^,^5^. When light strikes photochemical II (PSII) in the thylakoid membrane, water is disassembled into oxygen, electrons and protons. The protons then flow to photochemical I through the quinone molecule and cytochrome b6f, resulting in the synthesis of NADPH and ATP for carbohydrate synthesis from carbon dioxide. Excessive light energy and active oxygen species may damage the chloroplasts under conditions of light stress, leading to inhibition of growth (photoinhibition)^3^,^6^,^7^,^8^. Chloroplasts use ascorbate in these metabolic processes to eliminate active oxygen produced by electron transmission of PSII for the synthesis of NADPH in the stroma and as a coenzyme of violaxanthin de-epoxidase (VDE), which is involved in the release of photoenergy by heat dissipation in the xanthophyll cycle^4^,^6^,^7^,^8^. However, the mechanism by which ascorbate, which is synthesized in the mitochondria, passes through the envelope and thylakoid membranes of the chloroplast is poorly understood^9^. Although biochemical analyses indicated that the envelope membrane possesses a transporter that interacts preferentially with the reduced rather than the oxidized form of ascorbate (dehydroascorbate) as a transport substrate^9^,^10^, it is yet to be identified.

The SLC17 transporter family of *Arabidopsis* was originally reported as the Na^+^ or H^+^/phosphate co-transporter (PHT4) family consisting of six genes^11^. Although the PHT4 family is widely distributed in plants, including rice, poplar, *Physcomitrella patens* subsp. californica, and so on, as well as *Arabidopsis*, the physiological relevance of this family is unknown. Gene expression-profiling studies indicated that *AtPHT4;1*, *AtPHT4;4* and *AtPHT4;5* genes are strongly expressed in the leaves, *AtPHT4;3* and *AtPHT4;6* genes are expressed in both roots and leaves, and the *AtPHT4;2* gene is abundantly expressed in the roots^11^,^12^. Among these genes, only *AtPHT4;1* and *AtPHT4;4* showed ~10-fold increases in expression on light exposure^12^. On the other hand, as the levels of expression of all *AtPHT4s* changed little even under conditions of phosphorus deficiency, they were assumed to have functions in addition to their roles as phosphate transporters^11^.

A series of studies performed in our laboratory as well as those reported by other groups indicated that the mammalian SLC17 transporter family consists of nine members, which were shown to be membrane potential (Δψ)- and Cl^−^-dependent organic anion transporters: SLC17A1–2 act as urate exporters at the apical membranes of renal proximal tubules, SLC17A4 acts as a urate exporter at the apical membranes of intestinal ducts, SLC17A5 acts as a vesicular excitatory amino-acid transporter in synaptic vesicles, SLC17A6–8 act as vesicular glutamate transporters in synaptic vesicles, and SLC17A9 acts as a vesicular nucleotide transporter in synaptic vesicles and secretory granules^13^,^14^,^15^. The substrate specificity of each transporter is achieved by slight differences in amino-acid residues around the active centre: SLC17A1–2 and 4 transport urate, SLC17A5 transports aspartate and glutamate, SLC17A6–8 transport glutamate and SLC17A9 transports nucleotides^13^,^14^,^15^. On the basis of the above findings, we hypothesized that members of the AtPHT4 family also function as Δψ-dependent organic anion transporters, and that at least one of these proteins transports ascorbate anions.

The results of the present study indicate that AtPHT4;4 encodes an ascorbate transporter expressed at the envelope membranes of chloroplasts. In addition, both the levels of the reduced form of ascorbate in the leaves and the process of heat dissipation of excessive energy during photosynthesis are decreased in *Arabidopsis thaliana pht4;4* (*atpht4;4*) gene knockout mutants.

## Results

### Identification of an ascorbate transporter

**Figure 1:**
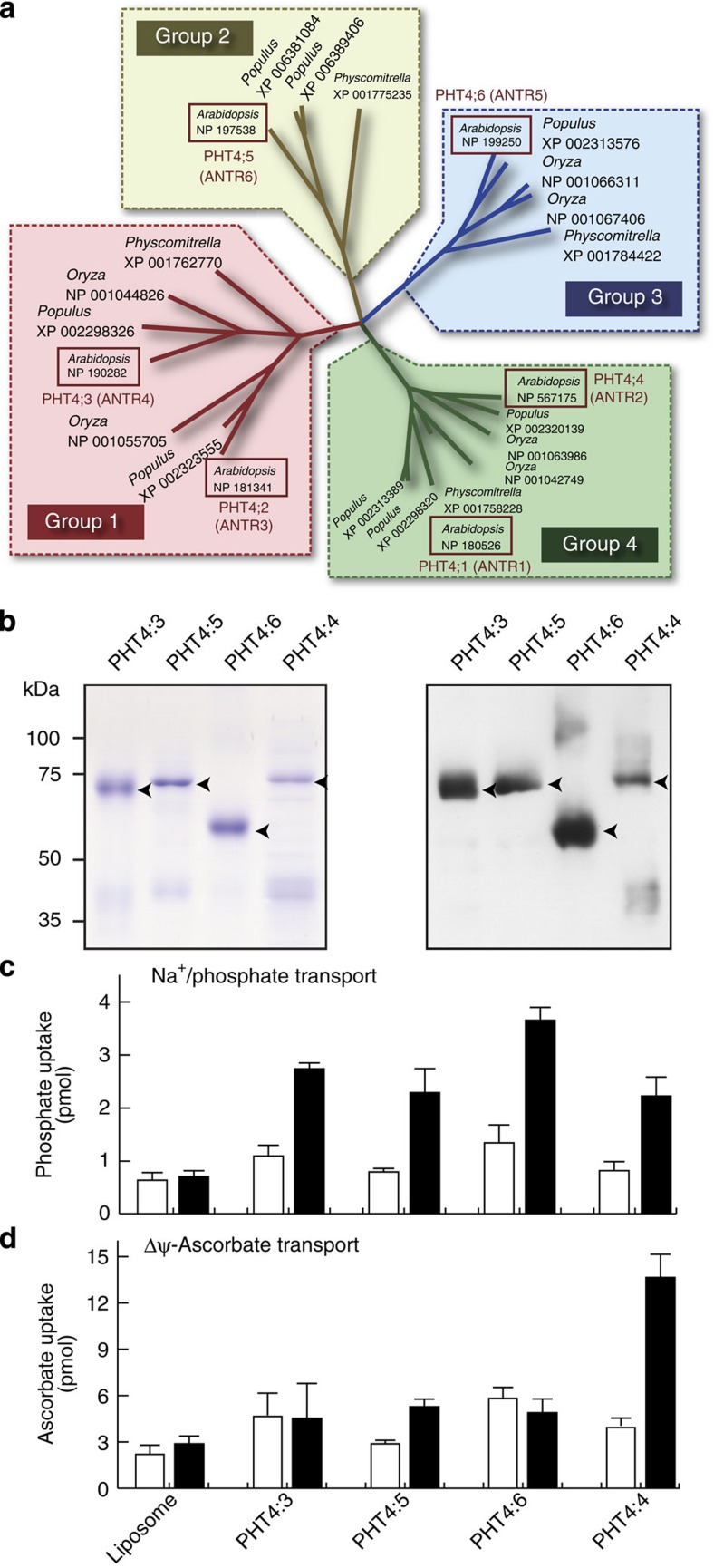
Phylogenetic tree of the plant SLC17 transporter family and ascorbate transporter of *Arabidopsis* SLC17 transporter family. (**a**) Phylogenetic tree of the plant SLC17 transporter family. *Arabidopsis* SLC17 transporters are indicated in red boxes. (**b**) Purification of *Arabidopsis* SLC17 transporter family. (Left) The purified fraction (10 μg) was analysed by 10% SDS-PAGE and visualized by CBB staining. (Right) A duplicate gel was analysed by immunoblotting with anti-6 × His antibody. The positions of marker proteins are indicated on the left. The positions of recombinant proteins are indicated by arrowheads. (**c**) Na^+^/P_i_ uptake by proteoliposomes containing purified AtPHT4 proteins at 2 min. ΔNa^+^-driven P_i_ uptake by proteoliposomes was assayed in the presence (closed bars) or absence (open bars) of Na^+^. (**d**) Ascorbate uptake by the proteoliposomes at 2 min. Δψ-driven ascorbate uptake by proteoliposomes was assayed in the presence (closed bars) or absence (open bars) of 2 μM valinomycin. Data are means±s.e., *n*=3–6.

The PHT4 family can be classified into four groups according to amino-acid sequence homology (Fig. 1a). To identify the ascorbate transporter from the PHT4 family, we selected one gene from each subgroup of the *Arabidopsis PHT4* family (subgroup 1: *AtPHT4;3*, subgroup 2: *AtPHT4;5*, subgroup 3: *AtPHT4;6*, and subgroup 4: *AtPHT4;4*), and their cDNAs were cloned into *Escherichia coli* expression vectors with a His-tag and soluble α-helix protein (β) coupled to both ends^16^. Each transporter was overexpressed in *E. coli*, solubilized and purified using Ni-NTA affinity column chromatography. The purified proteins were then electrophoresed and stained with Coomassie Brilliant Blue (Fig. 1b left), and their immunological properties were confirmed by immunoblotting with anti-6 × His antibodies (Fig. 1b right). The final fractions contained the major protein bands of the expected apparent molecular masses and immunological properties (Fig. 1b). These purified proteins were incorporated into proteoliposomes. By analogy to mammalian SLC17 family transporters, we investigated whether the transporters possess Na^+^-dependent transport activity for inorganic phosphate (P_i_). The Na^+^/P_i_ transport activity was detected in proteoliposomes containing all of these transporters, supporting the suggestion that all of the purified recombinant transporters were active in nature (Fig. 1c). Using the same batch of proteoliposomes, we employed Δψ (positive-inside) by addition of valinomycin in the presence of K^+^. The proteoliposomes established an inside-positive Δψ of ~90 mV through K^+^ diffusion, as reported previously^17^. Under these conditions, only proteoliposomes containing purified AtPHT4;4 exhibited significant ascorbate uptake activity, while those containing AtPHT4;3 or AtPHT4;6 did not (Fig. 1d). Proteoliposomes containing purified AtPHT4;5 exhibited slightly Δψ-dependent ascorbate uptake activity.

### Characterization of AtPHT4;4-mediated ascorbate uptake

**Figure 2:**
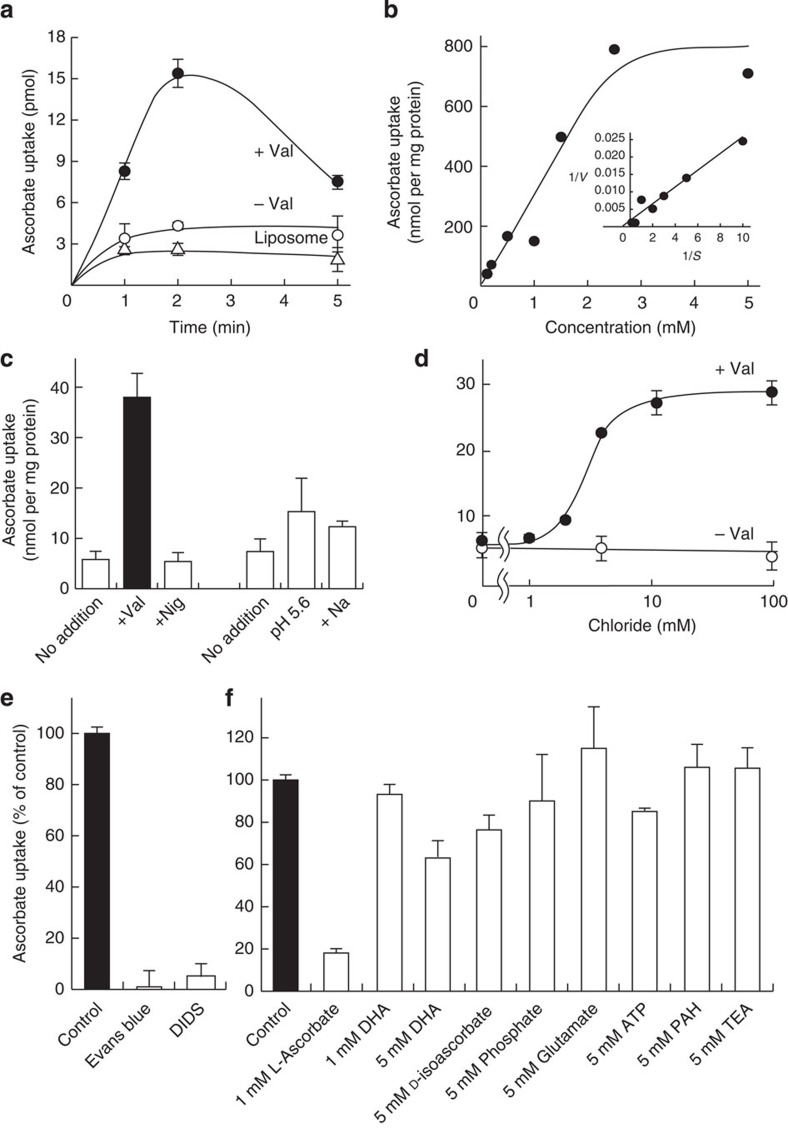
Characterization of ascorbate transport by AtPHT4;4. Proteoliposomes containing purified AtPHT4;4 were prepared, and ascorbate uptake was initiated by addition of 2 μM valinomycin. (**a**) Time course of proteoliposomes containing AtPHT4;4 in the presence (closed circles) or absence (open circles) of valinomycin, or no AtPHT4;4 in the presence of valinomycin (open triangles). (**b**) Dose dependence. The Δψ-dependent ascorbate uptake at 1 min was determined at various ascorbate concentrations. A Lineweaver–Burk plot is shown in the *inset.* (**c**) Driving force. Proteoliposomes containing Na^+^ or K^+^ were prepared and incubated in buffer containing K^+^ as indicated. Ascorbate uptake was measured at 2 min after addition of 2 μM valinomycin (Val) or 2 μM nigericin (Nig). For some experiments, proteoliposomes were prepared at pH 7.0 and K^+^, incubated in buffer at either pH 7.0 and K^+^, pH 5.6 and K^+^, or pH 7.0 and Na^+^, and assayed after 2 min. (**d**) Ascorbate uptake at 1 min was assayed in the presence or absence of different concentrations of Cl^−^. (**e**) The effects of inhibitors of ascorbate uptake at 1 min. The effects of Evans blue and 4,4′-diisothiocyano-2,2′-stilbenedisulphonic acid (DIDS), at 1 and 10 μM, were examined. (**f**) *cis*-Inhibition of ascorbate uptake at 1 min. AtPHT4;4-mediated uptake of 100 μM ascorbate was measured in the absence or presence of the listed compounds. Data are means±s.e., *n*=3–4.

We further characterized the ascorbate uptake by proteoliposomes containing purified AtPHT4;4. Valinomycin-induced Δψ was maximal at 1–2 min after addition of valinomycin (Supplementary Fig. 1). In parallel with the degree of Δψ formed, proteoliposomes showed maximal ascorbate uptake at 2 min, which decreased gradually thereafter (Fig. 2a). Liposomes lacking AtPHT4;4 showed only background uptake level. The Δψ-dependent ascorbate uptake exhibited Michaelis–Menten-type kinetics with *K*m and *V*max of 1.2 mM and 520 nmol min^−1^ mg^−1^, respectively (Fig. 2b). Bioenergetics analysis under conditions of defined Δψ, ΔpH and/or ΔpNa^+^ indicated that Δψ primarily triggered ascorbate uptake, while ΔpH and ΔpNa^+^ did not (Fig. 2c). Imposing ΔpH (outside-acidic) had a slight effect. Ascorbate uptake showed an absolute requirement for Cl^−^ similar to mammalian SLC17 family transporters^13^,^14^,^15^, and no ascorbate uptake was detected in the absence of Cl^−^. Ascorbate uptake showed marked activation with 2–4 mM Cl^−^ and reached a plateau at 10 mM Cl^−^ (Fig. 2d). Both Evans blue and 4,4′-diisothiocyano-2,2′-stilbenedisulphonic acid, which are typical inhibitors of mammalian SLC17 family transporters, inhibited ascorbate uptake (Fig. 2e)^13^,^14^,^15^. Experiments were performed to examine the effects of a spectrum of possible *cis*-inhibitors, and the results indicated that Δψ-dependent L-ascorbate uptake was insensitive to dehydroascorbate(oxidized L-ascorbate), D-isoascorbate (a stereoisomer of L-ascorbate), P_i_, glutamate, ATP, *p*-aminohippuric acid (PAH, a typical substrate of mammalian organic anion transporter) and tetraethylammonium (a typical substrate of mammalian organic cation transporters; Fig. 2f).

### Expression and localization of AtPHT4;4 in leaves

Quantitative PCR was performed to examine the level of *AtPHT4;4* gene expression. Consistent with previous observations^12^, the *AtPHT4;4* gene was expressed at higher levels in the leaves than the roots (Supplementary Fig. 2a), and its level of expression increased on light exposure (Supplementary Fig. 2b).

**Figure 3:**
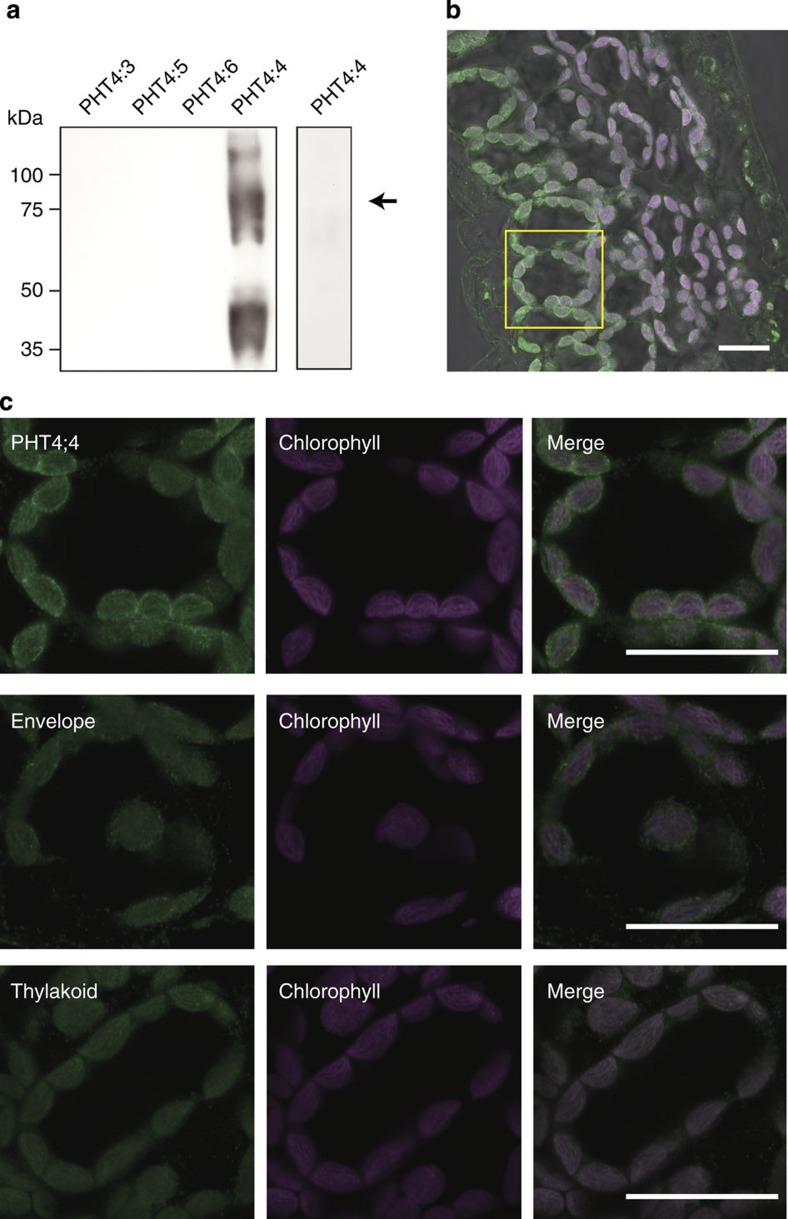
Expression of AtPHT4;4 protein and its association with the chloroplast envelope. (**a**) Immunological specificity of anti-AtPHT4;4 antibody. (Left) In a parallel experiment to that shown in Fig. 1b, immunoblotting analysis with anti-AtPHT4;4 was conducted. (Right) Preabsorbed antibodies were used as controls. The positions of marker proteins are indicated on the left. The position of AtPHT4;4 protein is indicated by an arrow. (**b**) Immunohistochemical localization of AtPHT4;4 in leaves. The fluorescence signals of AtPHT4;4 and chlorophyll are shown in green and magenta, respectively. Bar=20 μm. (**c**) (Upper) Higher magnification view of **b** (yellow box). (Middle and Lower) Merge of anti-TIC40 (envelope membrane marker) and chlorophyll, and anti-LHC2 (thylakoid membrane marker) and chlorophyll. Bar=20 μm.

We prepared a specific polyclonal antibody against AtPHT4;4 to examine its expression and localization. In a parallel experiment shown in Fig. 1b, the polyclonal antibody detected the AtPHT4;4 protein but not AtPHT4;3, AtPHT4;5 or AtPHT4;6 (Fig. 3a left), while pre-absorbed anti-AtPHT4;4 antibody did not bind to the AtPHT4;4 protein (Fig. 3a *right*) indicating the immunological specificity of the antibody for AtPHT4;4. On indirect immunofluorescence microscopy with the antibody, AtPHT4;4 immunoreactivity was detected in chloroplasts of the palisade tissue rather than spongy tissue from the leaves of *Arabidopsis* (Fig. 3b). Examination at higher magnification indicated that the AtPHT4;4 signal was present outside chlorophyll (Fig. 3c upper). The pattern of AtPHT4;4 localization was very similar to that of TIC40, which is an envelope membrane marker, but not light-harvesting chlorophyll protein 2 (LHC2), which is a thylakoid membrane marker (Fig. 3c middle and lower, respectively).

### *AtPHT4;4* gene knockout decreases reduced ascorbate in leaves

**Figure 4:**
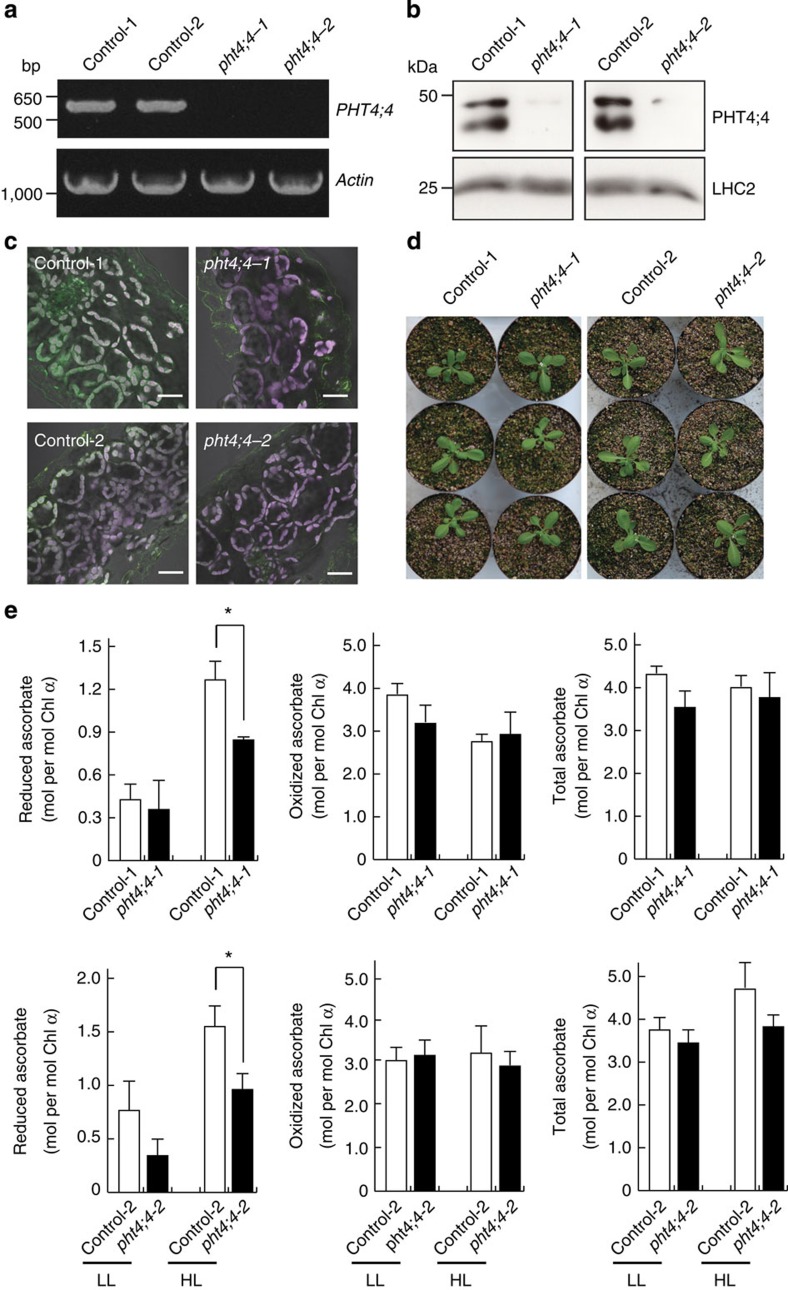
Ascorbate content was decreased in the leaves of *atpht4;4* mutants. (**a**) RT–PCR analysis was performed with total RNA isolated from leaves of control plants (Control-1 and Control-2) and mutants (*pht4;4-1* and *pht4;4-2*) using primers specific for *AtPHT4;4* and *AtA*ctin*2* mRNAs. (**b**) Immunoblotting was performed with crude membranes (50 μg) of chloroplasts prepared from four *Arabidopsis* lines using antibodies specific to AtPHT4;4 and LHC2 proteins. (**c**) Immunohistochemical expression of AtPHT4;4 in the leaves of four *Arabidopsis* lines. The fluorescent signals of AtPHT4;4 and chlorophyll are shown in green and magenta, respectively. Bar=20 μm. (**d**) Growth of the four *Arabidopsis* lines. Plants were grown under low-light conditions with a 16-h light/8-h dark cycle. Plants were photographed at the age of 4 weeks. (**e**) Contents of the reduced and oxidized forms of ascorbate in the leaves of control plants (Control-1 and Control-2, open bars) and mutants (*pht4;4-1* and *pht4;4-2*, closed bars) before (LL) and after (HL) transfer from low-light to high-light conditions (540 μmol photons m^−2^ s^−1^) for 2 min following 15-min dark adaptation. Total ascorbate is the sum of reduced and oxidized forms. Data are means±s.e., *n*=4–6, *P<0.05, Student’s *t*-test. Chl *a*, Chlorophyll *a*.

Two lines (ET4970; *pht4;4-1* and GT5039; *pht4;4-2*) of *Ds* transposon-tagged *atpht4;4* mutants were obtained from the Cold Spring Harbor Laboratory. Disruption of the *AtPHT4;4* gene in both mutant lines was confirmed using RT–PCR (Fig. 4a). The loss of the AtPHT4;4 protein in chloroplasts of both mutant lines was confirmed by immunoblotting and immunofluorescence microscopy (Fig. 4b,c). On microscopic observation, no morphological differences were detected between wild-type control and mutant lines (Fig. 4c). The *atpht4;4* mutant lines had a normal appearance compared with wild-type controls (that is, maximum rosette radius: 22.9±1.4, 20.8±1.6 and 24.0±0.7, 24.1±1.5 mm for control-1, *pht4;4-1* and control-2, *pht4;4-2*, respectively; Fig. 4d). Although the mutants were exposed to high light (300 μmol photons m^−2^ s^−1^) following initial growth under conditions of low light (100 μmol photons m^−2^ s^−1^), there were no significant changes in appearance compared with wild-type controls. Moreover, we measured the levels of ascorbate in the leaves of wild-type controls and *atpht4:4* mutant lines before and after exposure to high light stress. The levels of the reduced form of ascorbate in wild-type control leaves were increased by high light, whereas those of the reduced form of ascorbate in the *atpht4:4* mutant leaves under conditions of high light were decreased by ~35% compared with wild-type controls (Fig. 4e). On the other hand, no significant changes were observed in oxidized ascorbate levels between wild-type control and mutant leaves. Total ascorbate level was slightly decreased in the mutant leaves (Fig. 4e). Total ascorbate in the fraction containing chloroplasts from mutants was reduced to ~70% of that in wild-type controls (Supplementary Fig. 3).

### *atpht4;4* mutant lines are defective in the xanthophyll cycle

**Figure 5:**
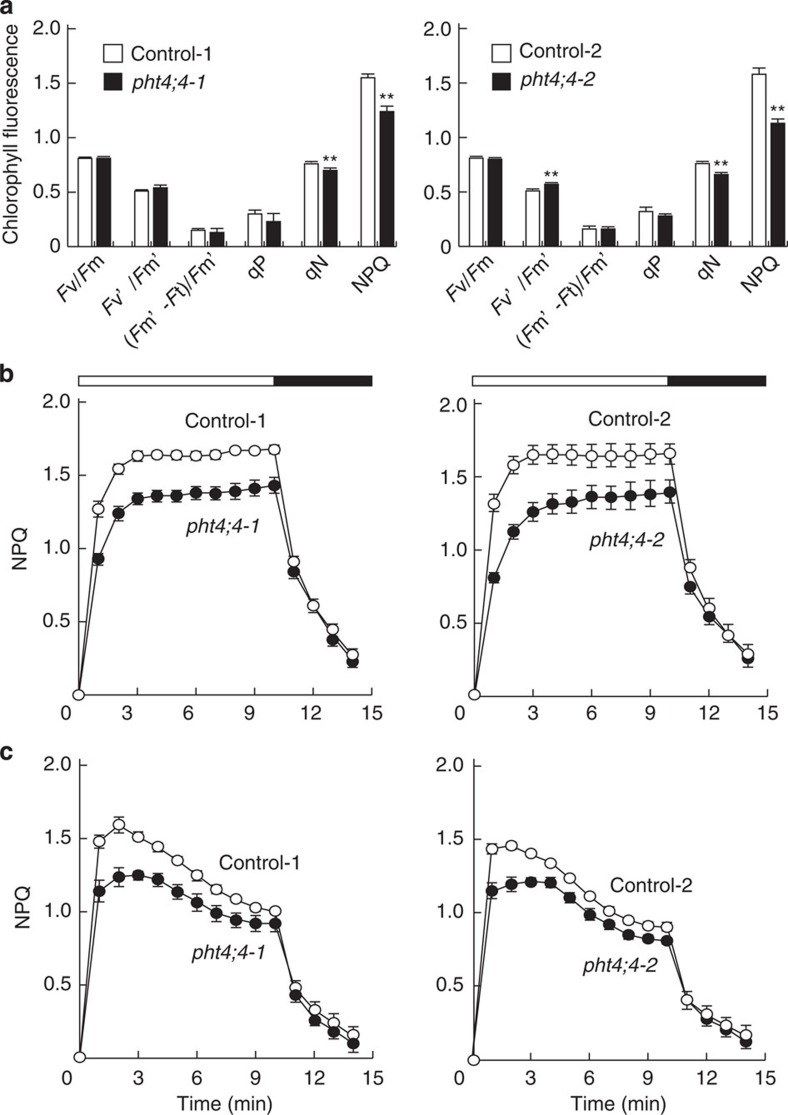
*atpht4;4* mutants showed decreased protection from excess absorbed light energy through thermal dissipation. (**a**) Chlorophyll fluorescence was measured in the leaves of control plants (Control-1 and Control-2, open bars) and mutants (*pht4;4-1* and *pht4;4-2*, closed bars) during 2 min of illumination with high light (HL, 540 μmol photons m^−2^ s^−1^). (**b**) NPQ was measured in the leaves of control plants (Control-1 and Control-2, open circles) and mutants (*pht4;4-1* and *pht4;4-2*, closed circles) during 10 min of illumination with HL (540 μmol photons m^−2^ s^−1^, open bars), followed by 4 min of darkness (closed bars). (**c**) NPQ was measured in the leaves of four *Arabidopsis* lines during 10 min of illumination with HL (230 μmol photons m^−2^ s^−1^, open bars), followed by 4 min of darkness (closed bars). Data are means±s.e., *n*=4, **P<0.01, Student’s *t*-test.

The fluorescence of chlorophyll was measured in the leaves of wild-type control and *atpht4;4* mutant lines. When illuminated with 540 μmol photons m^−2^ s^−1^, both mutant lines showed decreases in nonphotochemical quenching (NPQ and qN), corresponding to the dissipation of excess absorbed light energy as heat, but not *F*v/*F*m (the maximum quantum efficiency of PSII), *F*v′/*F*m′ (the efficiency of open reaction centre in light), (*F*m′–*F*t)/ *F*mF (the quantum yield of electron transfer at PSII) or photochemical quenching (the redox state of the primary quinone acceptor of PSII; Fig. 5a). The NPQ induction curves were compared between the two *atpht4;4* mutant lines (Fig. 5b,c). When illuminated at 540 μmol photons m^−2^ s^−1^ (Fig. 5b) or 230 μmol photons m^−2^ s^−1^ (Fig. 5c), the wild-type controls showed rapid establishment of NPQ within 2 min. In the *atpht4;4* mutants, however, NPQ was always ~20% lower than that in wild-type controls, although the level was similar to that in the wild-type controls in the dark period.

**Figure 6:**
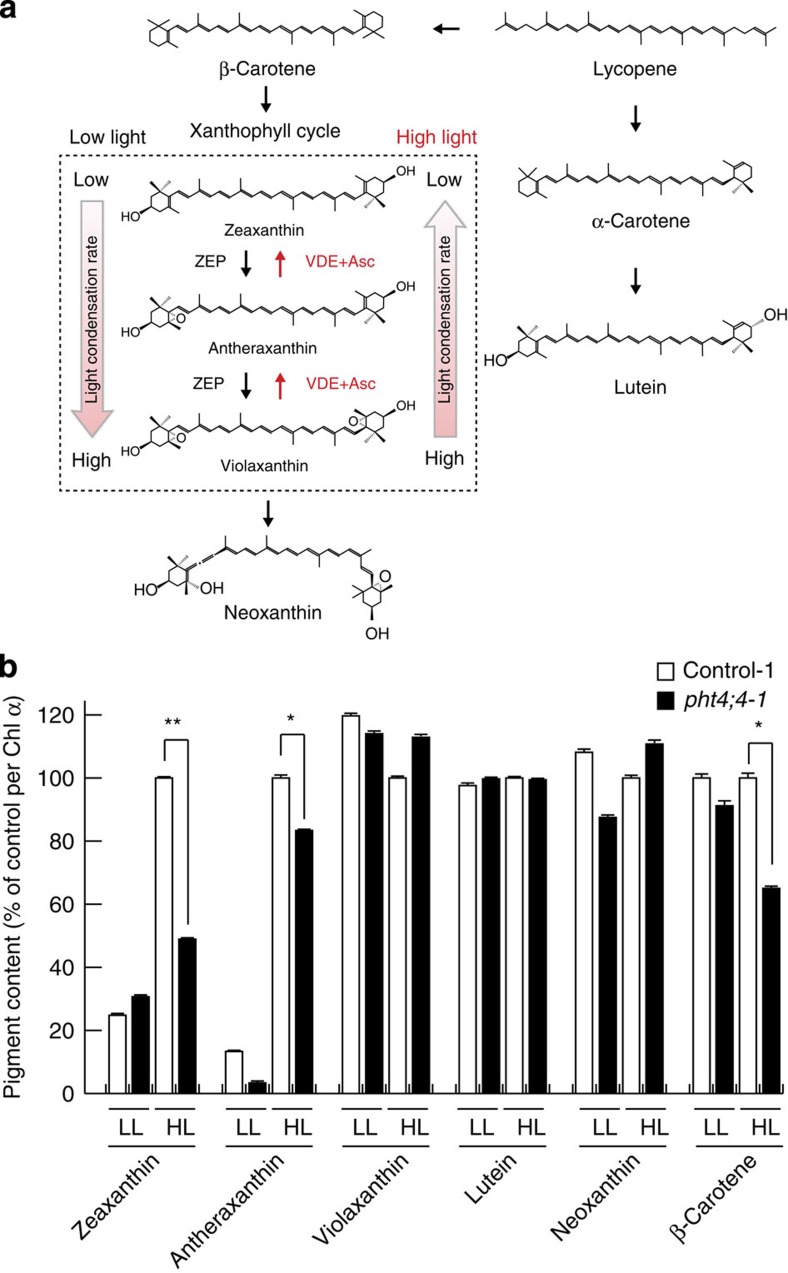
Pigment contents of wild-type and *atpht4;4* mutants. (**a**) Schematic model of xanthophyll cycle. Xanthophylls consist of three pigments—violaxanthin, antheraxanthin and zeaxanthin (dotted box). Under high-light conditions, VDE and ascorbate convert violaxanthin at a higher light condensation rate to antheraxanthin and then zeaxanthin at a lower rate with release of excessive light energy by heat dissipation. In contrast, zeaxanthin epoxidase (ZEP) converts zeaxanthin to antheraxanthin and then violaxanthin under low-light condition, leading to an increase in light condensation rate. (**b**) Pigment measurements in the leaves of wild-type (*wild-type1*, open bars) and mutants (*pht4;4-1*, closed bars) were performed before (LL) and after (HL) transfer from low light to high light (540 μmol photons m^−2^ s^−1^) for 2 min following 15-min dark adaptation. Control contents (100%) correspond to 13.7, 3.0, 26.4, 104.2, 34.4 and 34.0 mmol per mol Chl *a*, respectively. Data are means±s.e., *n*=10–12, *P<0.05, **P<0.01, Student’s *t*-test. Chl *a*, Chlorophyll *a*.

NPQ is a process by which xanthophylls, accessory pigments of LHC2, convert violaxanthin at a higher light-condensation rate to antheraxanthin and then zeaxanthin at a lower rate in order by VDE releasing excessive light energy by heat dissipation^6^,^7^,^8^. As ascorbate functions as a coenzyme of VDE, the xanthophylls and other pigments of wild-type and *atpht4;4* mutants were assayed by high-performance liquid chromatography (HPLC; Fig. 6a). The levels of zeaxanthin and antheraxanthin in the mutants were decreased by high-light treatment for 2 min, whereas the level of violaxanthin increased slightly (Fig. 6b, Supplementary Fig. 4). This is the first study to detect changes of xanthophyll cycle activities *in vivo* with short-term illumination for 2 min. There were no changes in the levels of lutein (constitutional isomer of zeaxanthin) or neoxanthin (one of the products of violaxanthin), neither of which is involved in NPQ (Fig. 6b, Supplementary Fig. 4). The above findings indicated that the levels of products of VDE using ascorbate as a coenzyme were decreased in the *atpht4;4* mutants. With regard to pigments other than xanthophylls, the levels of β-carotene were decreased by ~30% with high-light exposure in comparison with wild-type controls (Fig. 6b, Supplementary Fig. 4).

## Discussion

Previous efforts to identify ascorbate transporters and elucidate their physiological relevance in the plant kingdom have been unsuccessful. In the present study, we found that AtPHT4;4 transports the reduced form of L-ascorbate in a Δψ- and Cl^−^-dependent manner. AtPHT4;4 is abundantly expressed in the chloroplasts and is localized at the envelope membranes. Knockout of the *AtPHT4;4* gene resulted in decreased levels of the reduced form of ascorbate and a decrease in the xanthophyll cycle for heat dissipation of excessive energy in photosynthesis. These observations indicated that the AtPHT4;4 protein is an ascorbate transporter that is localized at the chloroplast envelope and may be required for photoinhibition tolerance. This is the first report regarding the identification of an ascorbate transporter in plants.

AtPHT4;4 exhibited Na^+^/P_i_ co-transport activity, similar to the mammalian SLC17 family transporters. As chloroplasts do not possess a Na^+^ gradient as a driving force, this activity is considered to be an evolutionarily conserved function of the ancestor protein. In addition, AtPHT4;4 also shows Cl^−^-dependent ascorbate-transport activity making use of Δψ as the driving force (Fig. 2)^13^,^14^,^15^. Although plants have evolved differently and have different transport substrates from mammals for the SLC17 transporter family, the fundamental transport mechanisms, such as Cl^−^ and Δψ dependence, have been preserved (Supplementary Fig. 5). The results of amino-acid sequence comparison indicated conservation of Arg230 in the fourth transmembrane domain of AtPHT4;4 in almost all SLC17 family transporters in mammals and *Arabidopsis* (Supplementary Figs 6 and 7). Our previous biochemical analyses of mammalian SLC17 family transporters by site-directed mutagenesis and homology modelling with bacterial glycerol 3-phosphate transporter indicated that this arginine residue is essential for Δψ-dependent organic anion transport activity and that the second and fourth transmembrane domains are important for binding to the transport substrate (Supplementary Fig. 7)^13^,^14^,^15^. The small differences in amino-acid residue around this domain are thought to determine the transport substrate specificity within the SLC17 transporter family. In addition, the amino-acid residues around this domain in AtPHT4;4 are also thought to be important in transport of the reduced form of L-ascorbate.

AtPHT4;4 is expressed more abundantly in the envelope membranes of chloroplasts of the palisade tissue, which is exposed to stronger photostress than the spongy tissue (Fig. 3b,c), suggesting that AtPHT4;4 may play a role in the transport of ascorbate into the chloroplast. The quantity of ascorbate transported to the stroma of the chloroplast is assumed to be controlled by Δψ as the driving force of AtPHT4;4, that is, the concentration gradient of ascorbate is dependent on the Δψ gradient. Although the chloroplast contains abundant ascorbate, its concentration gradient in the chloroplast and cytosol does not vary greatly because Δψ at the envelope of the chloroplast is small under physiological conditions^18^. The substrate specificity of ascorbate transport at the envelope membrane of the chloroplast was almost identical to that of AtPHT4;4 (refs 9, 10). Moreover, the level of the reduced form of ascorbate in the leaves in *Arabidopsis* mutants with a defect in the *AtPHT4;4* gene was ~35% lower than that in wild-type controls, almost corresponding to the content of ascorbate in the chloroplast (25–30% of the whole, Fig. 4e)^9^. The ascorbate content was also decreased in the fraction containing the chloroplasts of mutant leaves (Supplementary Fig. 3). On the basis of these findings, we concluded that AtPHT4;4 transports ascorbate from the cytosol into the chloroplast.

**Figure 7:**
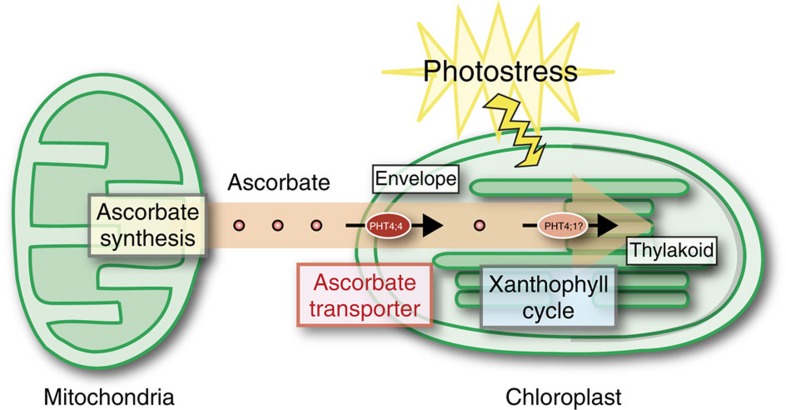
Schematic model of ascorbate transport in chloroplasts. Upon photostress, *PHT4;4* gene expression is enhanced, and the PHT4;4 protein at the envelope membranes takes up ascorbate from mitochondria, which is transferred into the thylakoid through an as yet unknown transporter. PHT4;1 is a candidate ascorbate transporter at the thylakoid membrane.

It should be noted that *AtPHT4;1* shows a high degree of identity with *AtPHT4;4* (identity of 70% at the amino-acid sequence level). Previous studies indicated that both *AtPHT4;1* and *AtPHT4;4* gene expression are induced by light^12^. AtPHT4;4 is present in the envelope membrane of the chloroplast, as shown in Fig. 3c, while AtPHT4;1 is localized at the thylakoid membrane of the chloroplast^19^,^20^. Thus, AtPHT4;1 is likely to also function as an ascorbate transporter at the thylakoid membrane. AtPHT4;4 transports ascorbate into the stroma of the chloroplast, and AtPHT4;1 transports ascorbate from the stroma to the thylakoids to control the dynamic state of ascorbate in the chloroplasts (Fig. 7). Further studies of the transport function and physiological role of PHT4;1 are currently in progress in our laboratory.

*AtPHT4;4* gene defect results in a decrease of ascorbate content in the chloroplasts. Although the *AtPHT4;4* gene defect was not associated with any visible phenotype, we found marked variations in the xanthophyll cycle during photosynthesis in the mutants. As VDE requires ascorbate as a coenzyme, this variation may have been due to the *AtPHT4;4* gene defect^6^,^7^. To date, two types of mutant lines with decreased NPQ in *Arabidopsis* have been reported, that is, ascorbate synthetic enzyme mutant lines (*vtc*) and VDE mutant lines (*npq1*)^4^,^21^. With regard to *vtc* mutant lines, four genes (*vtc1–4*) have been reported as variants that reduce the ascorbate levels in young leaves to ~30–50% of those in wild-type controls. Although NPQ in measurement of chlorophyll fluorescence was decreased in *vtc* mutant lines, there were no changes in other parameters, as observed in *atpht4;4* mutant lines. Moreover, it was also reported that *npq1* mutant lines showed decreased NPQ and zeaxanthin levels in the xanthophyll cycle as observed in *atpht4;4* mutant lines. Interestingly, there were no marked changes in growth in *vtc* or *npq1* mutant lines, and their β-carotene levels were decreased under conditions of high-light exposure, as observed in *atpht4;4* mutant lines^22^,^23^. The similarities in variations among the mutants of ascorbate synthetic enzyme, ascorbate transporter and VDE strongly support our conclusion.

Here we postulate a mechanism for the reduction in the level of β-carotene in these mutant lines from the viewpoint of antioxidant action as follows. Reactive oxygen species are produced during photosynthesis within the chloroplasts of plants and inhibit photosynthesis. Ascorbate has an antioxidant action for detoxification; active oxygen is converted to H_2_O_2_ in the stroma by superoxide dismutase and the H_2_O_2_ is then converted to H_2_O by ascorbate peroxidase^3^,^4^. On the other hand, β-carotene is an accessory pigment of LHC2, and it has been reported that β-carotene plays an important role in photosynthesis as an antioxidant^24^,^25^. β-Carotene nonenzymatically converts active oxygen species produced by photosynthesis to oxygen inside the thylakoid membrane for detoxification. Thus, in *atpht4;4* mutant lines, β-carotene is inferred to function as an antioxidant in place of ascorbate to protect against photoinhibition. In addition, it has been suggested that C3 plants, which only have the Calvin–Benson cycle in the carbon fixation reaction, consume oxygen and release carbon dioxide through photorespiration to prevent oxidation by high concentrations of oxygen under conditions of high-light exposure^26^. In *atpht4;4* mutant lines, as the levels of accessory pigments in the xanthophyll cycle are decreased, the level of light energy absorbed by plants under conditions of high-light exposure is assumed to be decreased as is the level of oxygen. Thus, excessive oxygen is not produced easily, resulting in a mitigation of oxidative stress. We speculate that these actions serve as an alternative mechanism of antioxidant action when the level of the reduced form of ascorbate inside the chloroplasts is decreased, which is a factor not observed in variations other than NPQ in *atpht4;4* mutant lines.

In summary, we identified the ascorbate transporter in the chloroplast envelope membrane in *Arabidopsis*, and demonstrated that it plays a role in the xanthophyll cycle during photosynthesis. Heritable transporter genetic modification technology may provide a means of developing photoinhibition-tolerant plants.

## Methods

### cDNA

cDNAs of *AtPHT4;3* (Accession No. NM_114565.2), *AtPHT4;5 (*Accession No. NM_122045.3), *AtPHT4;6* (Accession No. NM_123804.3) and *AtPHT4;4* (Accession No. NM_116261.4) were provided by the RIKEN BRC through the National Bio-Resource Project of the MEXT, Japan. The cDNAs were amplified by PCR with the following primer pairs: *AtPHT4;3*, forward: 5′-CGGGGGATCCGAATTCATGTGTTACTCTCTCTCTATAC-3′, reverse: 5′-CCTTGTTCATCTCGAGAGCTGTTGTGTCAAAATCTACT-3′; *AtPHT4;5*, forward: 5′-CGGGGGATCCGAATTCATGGCGAGACTTACCTTGAG-3′, reverse: 5′-CCTTGTTCATCTCGAGTGAGTCTTCCTTTCTGAACGT-3′; *AtPHT4;6*, forward: 5′-CGGGGGATCCGAATTCATGAAGTTATCAAATATTCCGC-3′, reverse: 5′-CCTTGTTCATCTCGAGATCAAAGATCCTTTCTCCAGT-3′; *AtPHT4;4*, forward: 5′-CGGGGGATCCGAATTCATGGCCCTCGGTGGCTTGAT-3′, reverse: 5′-CCTTGTTCATCTCGAGTTCGAGAATTTTTTCT-3′. The amplified DNA fragments were cloned into β-pET-28a(+)-β, an expression vector for eukaryotic membrane proteins in *E. coli*^16^, using the In-Fusion cloning kit (TaKaRa).

### Antibodies

Site-specific rabbit polyclonal antibody against AtPHT4;4 was prepared by repeatedly injecting glutathione *S*-transferase fusion polypeptides encoding M1-Q127 of AtPHT4;4 into a rabbit. Rabbit polyclonal anti-AtLhcb1 antibody (Agrisera, Catalogue No. AS01 004), rabbit polyclonal anti-AtTic40 antibody (Agrisera, Catalogue No. AS010 709), peroxidase-conjugated mouse monoclonal anti-His6 antibody (clone His-2; Roche, Catalogue No. 04 905 270 001) and Alexa Fluor 488 goat anti-rabbit IgG (Molecular Probes, Catalogue No. A-11008) were purchased from the sources shown.

### Expression and purification of AtPHT4

The expression and purification of AtPHT4 were carried out as described previously^16^. *E. coli* C43 (DE3) cells were transformed with expression vectors and grown in TB medium containing 20 μg ml^−1^ kanamycin sulphate at 37 °C. *E. coli* cells were grown until *A*_600_ reached 0.6–0.8, and then isopropyl-β-D-thiogalactopyranoside was added to a final concentration of 1 mM and culture was continued for 16 h at 18 °C. The cells were then harvested by centrifugation and suspended in a buffer containing 20 mM Tris–HCl (pH 8.0), 300 mM sucrose and 1 mM phenylmethylsulphonyl fluoride. The cell suspension was then disrupted by sonication with a TOMY UD200 tip sonifier (OUTPUT4), and centrifuged at 5,856 × *g* at 4 °C for 10 min to remove large inclusion bodies and cell debris. The resultant supernatant was carefully obtained and centrifuged again at 150,000 × *g* for 1 h at 4 °C. The pellet was suspended in buffer containing 70 mM Tris–HCl (pH 8.0), 100 mM NaCl, 10 mM KCl, 15% glycerol and 1 mM phenylmethylsulphonyl fluoride, and the protein concentration was adjusted to 10 mg ml^−1^. Then, the membranes were treated with 1.5% Fos-choline 14 (Affymetrix) and centrifuged at 150,000 × *g* at 4 °C for 1 h. The supernatant containing AtPHT4 was obtained, diluted twofold with buffer containing 70 mM Tris–HCl (pH 8.0), 100 mM NaCl, 10 mM KCl, 15% glycerol and 1 mM phenylmethylsulphonyl fluoride, and then applied to a column containing 1 ml of nickel-NTA Superflow resin (Qiagen) equilibrated with buffer containing 70 mM Tris–HCl (pH 8.0), 100 mM NaCl, 10 mM KCl and 15% glycerol. After incubation for 1 h at 4 °C, the column was washed with washing buffer containing 70 mM Tris–HCl (pH 8.0), 5 mM imidazole, 100 mM NaCl, 10 mM KCl, 20% glycerol and 0.1% *n*-decyl-β-D-thiomaltopyranoside (Affymetrix). The AtPHT4 protein was eluted with buffer containing 20 mM Tris–HCl (pH 8.0), 300 mM imidazole, 100 mM NaCl, 10 mM KCl, 20% glycerol and 0.1% *n*-decyl-β-D-thiomaltopyranoside, and then stored at −80 °C, at which it was stable without loss of activity for at least several months.

### Reconstitution

Aliquots of 20 μg of purified AtPHT4 were mixed with 500 μg of liposomes and frozen at −80 °C for at least 10 min. The mixture was diluted 60-fold with reconstitution buffer containing 20 mM MOPS-Tris (pH 7.0), 0.15 M sodium acetate and 5 mM magnesium acetate. The buffer composition was changed as necessary. Reconstituted proteoliposomes were pelleted by centrifugation at 200,000 × *g* for 1 h at 4 °C, and then suspended in 0.2 ml of reconstitution buffer. Asolectin liposomes were prepared as described previously^17^. Soybean lecithin (10 mg ml^−l^; Sigma Type IIS) was suspended in the buffer containing 20 mM MOPS-Tris (pH 7.0) and 1 mM dithiothreitol. The mixture was sonicated until clear in a bath-type sonicator, and stored at −80 °C until use.

### Transport assay

Transport assays were carried out by the gel permeation procedure as described previously^17^. Reaction mixtures (130 μl) containing 0.3 μg of protein incorporated into proteoliposomes, 20 mM MOPS-Tris (pH 7.0), 0.15 M potassium acetate, 5 mM magnesium acetate, 4 mM KCl, 2 μM valinomycin and 100 μM L-[1–^14^C] ascorbate (0.5 MBq μmol^−1^; PerkinElmer) were incubated at 27 °C. At the times indicated, transport was terminated by separating the proteoliposomes from the external medium using centrifuge columns containing Sephadex G-50 (fine). The radioactivity in the eluate was measured by liquid scintillation counting. In the case of Na^+^/P_i_ co-transport, the assay reaction mixture contained 0.3 μg of protein incorporated into proteoliposomes, 20 mM MOPS-Tris (pH 7.0), 5 mM magnesium acetate, 4 mM KCl, 0.1 M sodium acetate and 100 μM [^32^P] KH_2_PO_4_ (3.7 MBq μmol^−1^; PerkinElmer).

### Plant materials and growth conditions

Plants were germinated and grown in soil under well-watered conditions at 22 °C under a 16-h light/8-h dark cycle. The *atpht4;4-1* (ET4970) and *atpht4;4-2* (GT5039) mutants were *Ds* transposon-tagged mutants of the Landsberg ecotype, and were obtained from the Cold Spring Harbor Laboratory (New York). Control-1 and Control-2 are segregated lines without *Ds* insertion from ET4970 and GT5039, respectively. Genomic DNA of *Arabidopsis* plants was prepared using an automated DNA isolation system (PI-50alpha; Kurabo). To determine the genotype of *pht4;4-1* and *pht4;4-2*, PCR-based genotyping was performed with the following primers: PHT4-L2 (5′-ATGGAGATGCGTTCTGTAGATT-3′), PHT4-R (5′-GGTTCCAACGAGTAGAAGATGA-3′), Ds3–4 (5′-CCGTCCCGCAAGTTAAATATG-3′) and Ds5-3 (5′-TACCTCGGGTTCGAAATCGAT-3′). Total RNA from *Arabidopsis* plants was prepared for RT–PCR using an RNeasy Plant Mini Kit (Qiagen). RT–PCR was performed using a PrimeScript One-Step RT-PCR kit (Takara) with the primers PHT4_L2 and PHT4_R. As a loading control, Actin2 transcripts were amplified with the primers Actin2RT-F (5′-GACCTGCCTCATCATACTCG-3′) and Actin2RT-R (5′-TTCCTCAATCTCATCTTCTTCC-3′). Full-size gel images are shown in Supplementary Fig. 8a.

### Quantitative PCR

Total RNA was prepared from the leaves and roots of 4- to 5-week-old Columbia wild-type plants using an RNeasy Plant Mini Kit (Qiagen). cDNA was generated from total RNA with a PrimeScript RT reagent Kit (Takara) using 1 μg of total RNA as the template. Quantitative PCR was carried out with specific forward and reverse primers at 0.4 μM and 5 units μl^−1^ of SYBR Premix Ex Taq II (Takara). Reactions were performed for 35 cycles of denaturation at 95 °C for 15 s and annealing/extension at 60 °C for 30 s. The primer set used for detection of *AtPHT4;4* was as follows: 5′-TCGGGTCTCTACTCTAATCATCAAG-3′ and 5′-AACACATCATCCCATGAACCTCG-3′. The level of *AtPHT4;4* expression was evaluated relative to that of the housekeeping gene (*AtActin2*) by relative standard curve method using the StepOne Software v2.2.2 (Life Technologies). The primer set used for detection of *AtActin2* was as follows: 5′-CCATCCAAGCTGTTCTCTCCTTG-3′ and 5′-GGTAATCAGTAAGGTCACGTCCAG-3′.

### Immunohistochemistry

Immunostaining of AtPHT4;4 in leaves of 4- to 5-week-old plants was performed as described previously^27^. Leaves were fixed in 4% (w/v) paraformaldehyde and 60 mM sucrose buffered with 50 mM cacodylic acid (pH 7.4) for 2 h at room temperature with occasional degassing. After three washes with 60 mM sucrose and 50 mM cacodylic acid (pH 7.4), the fixed samples were embedded in 5% agar and cut into sections 80 μm thick with a microslicer (ZERO 1; Dosaka EM). Sections were placed on microscope slides, incubated with PBS (10 mM, pH 7.4, 138 mM NaCl, 2.7 mM KCl) containing 0.1% (w/v) pectolyase Y-23 (Seishin) at 30 °C for 2 h and then reincubated in PBS containing 0.3% (v/v) Triton X-100 at 30 °C for 2 h, washed three times with PBS and blocked with 5% (w/v) bovine serum albumin in PBS. Slides were incubated in a chamber at 37 °C with purified rabbit anti-AtPHT4;4 polyclonal antibody (1:500 dilution in PBS). After three washes in PBS and blocking with 5% (w/v) bovine serum albumin in PBS, the slides were exposed to secondary antibody (1:2,000 dilution in PBS, Alexa Fluor 488 goat anti-rabbit IgG) for 2 h at room temperature, washed five times in PBS and mounted with 50% (v/v) glycerol in PBS. Samples were examined with a laser-scanning confocal microscope (LSM510; Carl Zeiss).

### Preparation of chloroplasts for immunoblotting

Leaves of 4- to 5-week-old plants were homogenized with a biomasher III (Nippi) in buffer containing 20 mM MOPS-Tris (pH 7.0), 330 mM sorbitol, 0.2 mM MgCl_2_, 10 μg ml^−1^ pepstatin A and 10 μg ml^−1^ leupeptin. The extract was centrifuged at 500 × *g* at 4 °C for 1 min to remove cell debris. The resultant supernatant was carefully obtained and centrifuged again at 3,000 × *g* for 5 min at 4 °C. The pellet (chloroplast fraction) was suspended with the same buffer. The fraction (50 μg) was separated by 10% SDS–PAGE and analysed by immunoblotting with rabbit polyclonal antibodies against AtPHT4;4 (1:1,000 dilution) and AtLhcb1 (1:5,000 dilution)^17^. Full-size blot images are shown in Supplementary Fig. 8b.

### Measurement of ascorbate

Ascorbate levels were measured as described previously^28^,^29^. The same batch of chlorophyll was immediately frozen in liquid nitrogen. The frozen leaves were ground to a fine powder and extracted with 400 μl of 0.2 N HCl for 30 min. The extract was neutralized with 0.2 N NaOH and 0.2 M NaH_2_PO_4_ (pH 5.6), centrifuged at 16,000 × *g* for 10 min at 4 °C and the supernatants were pooled. Total ascorbate was determined by spectrophotometry to measure ultraviolet absorption by the reduced form of ascorbate at 265 nm (*A*_265_). In this assay, ascorbate oxidase was used to oxidize all ascorbate, and the amount of ascorbate was determined from the difference in *A*_265_ before and after addition of the enzyme. To obtain values for oxidized and total ascorbate, samples were reduced by addition of 5 mM dithiothreitol. The values were corrected relative to chlorophyll *a*.

### Extraction of ascorbate from chloroplast fraction

Plants were germinated and grown on MS medium containing 1% (wt/vol) sucrose and 0.8% agar in a growth chamber at 22 °C under a 16-h light/8-h dark cycle. Leaves of 2-week-old plants were sliced in buffer containing 20 mM MOPS-Tris (pH 7.0), 330 mM sorbitol, 0.2 mM MgCl_2_ and 0.2% BSA. The extract was passed through cell strainer with 40 μm nylon mesh (Falcon), and centrifuged at 500 × *g* at 4 °C for 1 min to remove cell debris. The resultant supernatant was carefully obtained and centrifuged at 3,000 × *g* for 5 min at 4 °C. The pellet (chloroplast fraction) was extracted with 200 μl of 0.2 N HCl for 10 min. The extract was neutralized with 2 N NaOH and 0.2 M NaH_2_PO_4_ (pH 5.6), centrifuged at 16,000 × *g* for 10 min at 4 °C and the supernatants were pooled.

### Measurement of chlorophyll fluorescence

Standard modulated chlorophyll fluorescence measurements were performed with 15-min dark-adapted plant leaves using a miniaturized pulse-amplitude-modulated photosynthesis yield analyser (PAM 101/103; Walz) as described previously^30^. Leaves of 4- to 5-week-old plants were subjected to a saturating light pulse and then illuminated (230 μmol photons m^−2^ s^−1^ or 540 μmol photons m^−2^ s^−1^) for 10 min followed by 4 min of darkness. NPQ was calculated as (*F*m−*F*m′)/*F*m′, where *F*m′ and *F*m are the maximum PS II fluorescence in the light-adapted state and the dark-adapted state, respectively.

### Measurement of pigments

Measurement of pigments was carried out as described previously^8^,^28^. The same batch of chlorophyll was immediately frozen in liquid nitrogen. The frozen leaves were ground to a fine powder and extracted with 150 μl of 80% (v/v) acetone by vortexing for 1 min. The extract was centrifuged at 16,000 × *g* for 10 min at 4 °C and the supernatant was saved. Another 150 μl of 80% (v/v) acetone was added to the pellet and mixed thoroughly. The extract was centrifuged again, and the supernatants were pooled. Aliquots of 50 μl of the supernatant were subjected to HPLC and separated on a Spherisorb S5 ODS 4.6 × 250 mm cartridge column (Waters) at 25 °C. Pigments were eluted with a linear gradient from 100% (v/v) solvent A (acetonitrile:methanol:0.1 M Tris–HCl, pH 8.0; 84:2:14 [v/v]) to 100% (v/v) solvent B (methanol:ethyl acetate, 68:32 [v/v]) for 15 min, followed by 3 min of solvent B. The solvent flow rate was 1.2 ml min^−1^. Pigments were detected by *A*_445_ with a reference at 550 nm by a diode array detector. The values were corrected relative to chlorophyll *a*.

### Data analysis

All numerical values are shown as the means±s.e.m.; *n*=3–12, unless otherwise specified. Statistical significance was determined by Student’s *t*-test. Significance was defined as *P<0.05 or **P<0.01.

## Author contributions

T.M., T.K., H.O. and Y.M. designed the experiments, analysed the data, wrote the paper and performed the experiments. Y.T., N.Y., K.Y., A.S. and E.S. performed the experiments and analysed the data. J.F.M. and K.S. designed the experiments, analysed the data and wrote the paper.

## Additional information

**How to cite this article:** Miyaji, T. *et al.* AtPHT4;4 is a chloroplast-localized ascorbate transporter in *Arabidopsis*. *Nat. Commun.* 6:5928 doi: 10.1038/ncomms6928 (2015).

## Supplementary Material

Supplementary Information Supplementary Figures 1-8
