# Supplementary material for: AtPHT4;4 is a chloroplast-localized ascorbate transporter in Arabidopsis
Source: Nat Commun. 2015 Jan 5;6:5928. doi: 10.1038/ncomms6928 (PMC4308718; doi:10.1038/ncomms6928)
Supplement: Supplementary Information — Supplementary Figures 1-8 [file ncomms6928-s1.pdf]

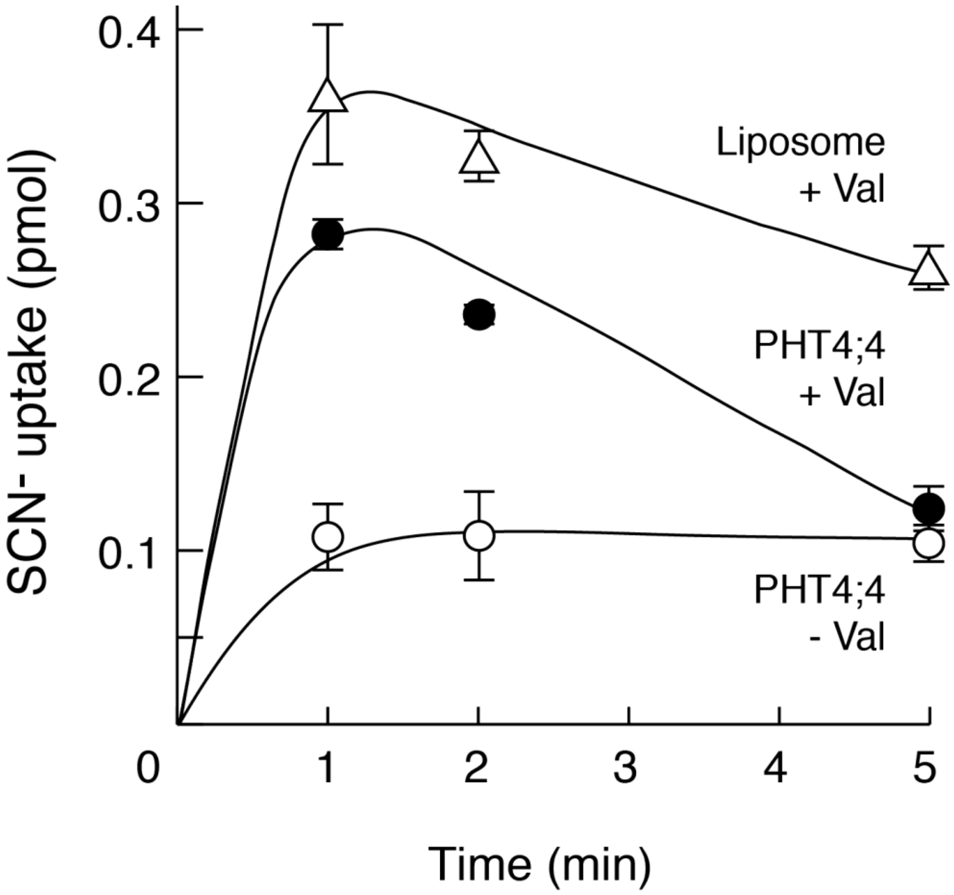

Supplementary Figure 1. Valinomycin-induced formation of Δψ.

Proteoliposomes containing purified AtPHT4;4 were incubated under assay conditions of ascorbate uptake in the presence of 20 μM [^{14}C] potassium SCN^- (membrane potential indicator). SCN^- uptake was initiated by the addition of 2 μM valinomycin. Time course of proteoliposomes containing AtPHT4;4 in the presence (closed circles) or absence (open circles) of valinomycin, or no AtPHT4;4 in the presence of valinomycin (open triangles). Data are means ± SE, *n* = 3.

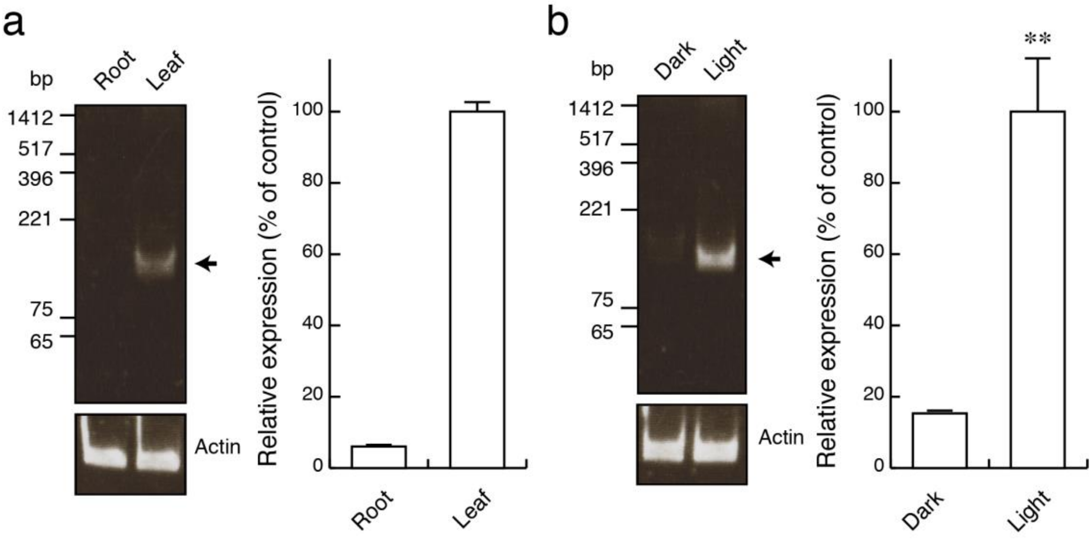

Supplementary Figure 2. *AtPHT4;4* gene expression and the effects of light exposure.

(**a**) *AtPHT4;4* gene expression in the leaves and roots of *Arabidopsis*. The expression was examined by quantitative PCR analysis with total RNA from the indicated organs using probes specific to *AtPHT4;4* or *AtA*ctin*2*. The resultant PCR products were analysed by electrophoresis. The arrows indicate the positions of *AtPHT4;4* (149 bp, *upper panel*) and *AtActin2* (162 bp, *lower panel*). The levels of *AtPHT4;4* mRNA were defined as ratios relative to those of *AtA*ctin*2*. (**b**) *AtPHT4;4* gene expression in the leaves in the presence or absence of light exposure for 5 hours. The expression was examined by quantitative PCR with total RNA using primers specific to *AtPHT4;4* and *AtA*ctin*2*. The resultant PCR products were analysed by electrophoresis. The levels of *AtPHT4;4* mRNA were defined as ratios relative to those of *AtA*ctin*2*. Data are means ± SE, $n = 4$, $^{**}P < 0.01$, Student's t-test.

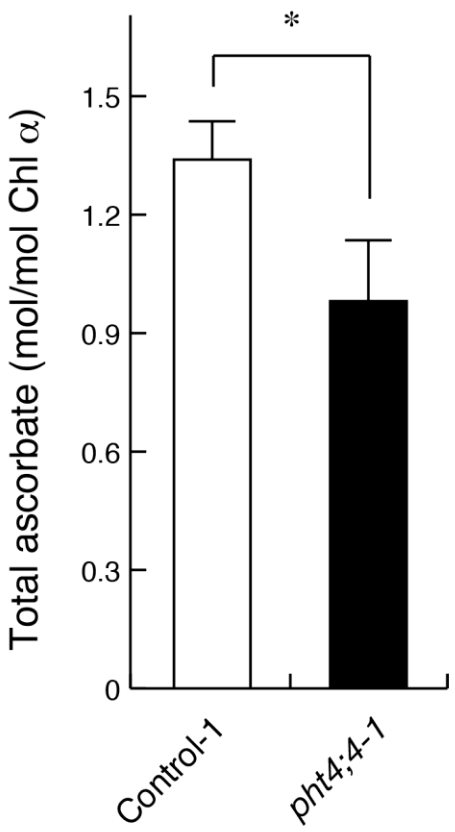

Supplementary Figure 3. The ascorbate content was decreased in isolated chloroplasts of *atpht4;4* mutants.

Total ascorbate contents in isolated chloroplasts of control plants (Control-1, open bars) and mutants (*pht4;4-1*, closed bars) under normal light condition. Data are means ± SE, $n = 5$, $^*P < 0.05$, Student's t-test. Chl *a*, Chlorophyll *a*.

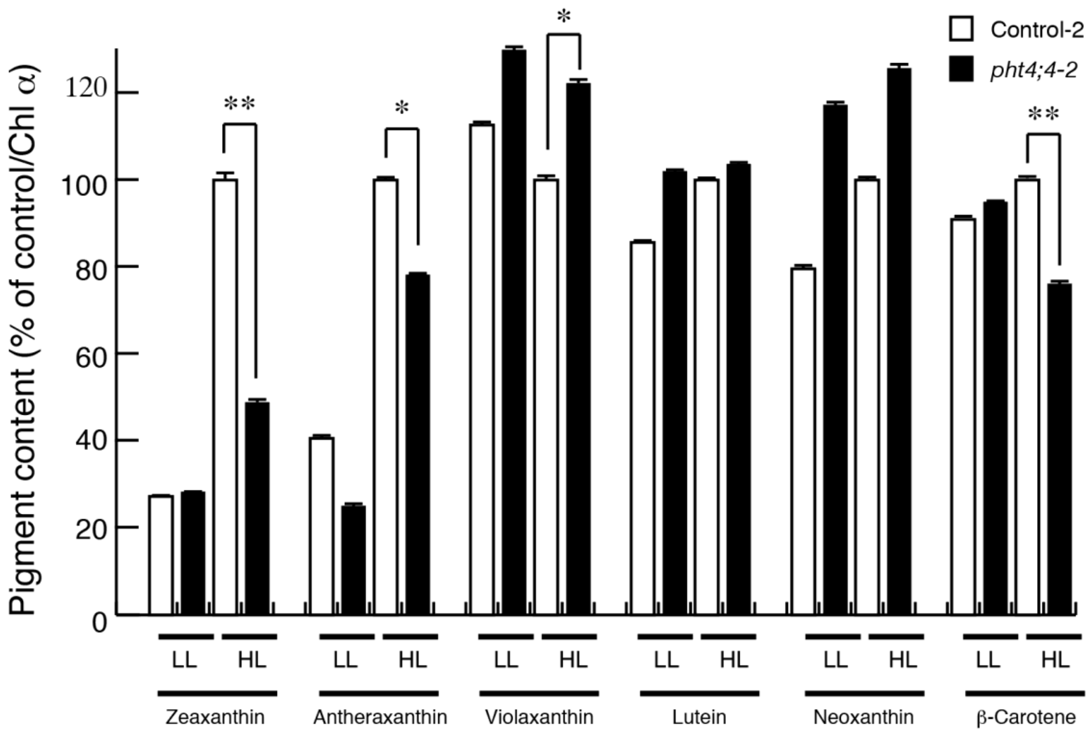

Supplementary Figure 4. Pigment contents of wild-type controls and *atpht4;4* mutants.

Pigment measurements in the leaves of control plants (Control-2, open bars) and mutants (*pht4;4-2*, closed bars) were performed before (LL) and after (HL) transfer from low light to high light (HL, 540 μmol photons $m^{-2} s^{-1}$) for 2 minutes following 15-minute dark-adaptation. Control contents (100%) correspond to 20.9, 3.2, 22.1, 108.9, 25.0, and 51.0 mmol per mol Chl *a*, respectively. Data are means ± SE, $n = 10 – 12$, *P<0.05, **P < 0.01, Student's t-test. Chl *a*, Chlorophyll *a*.

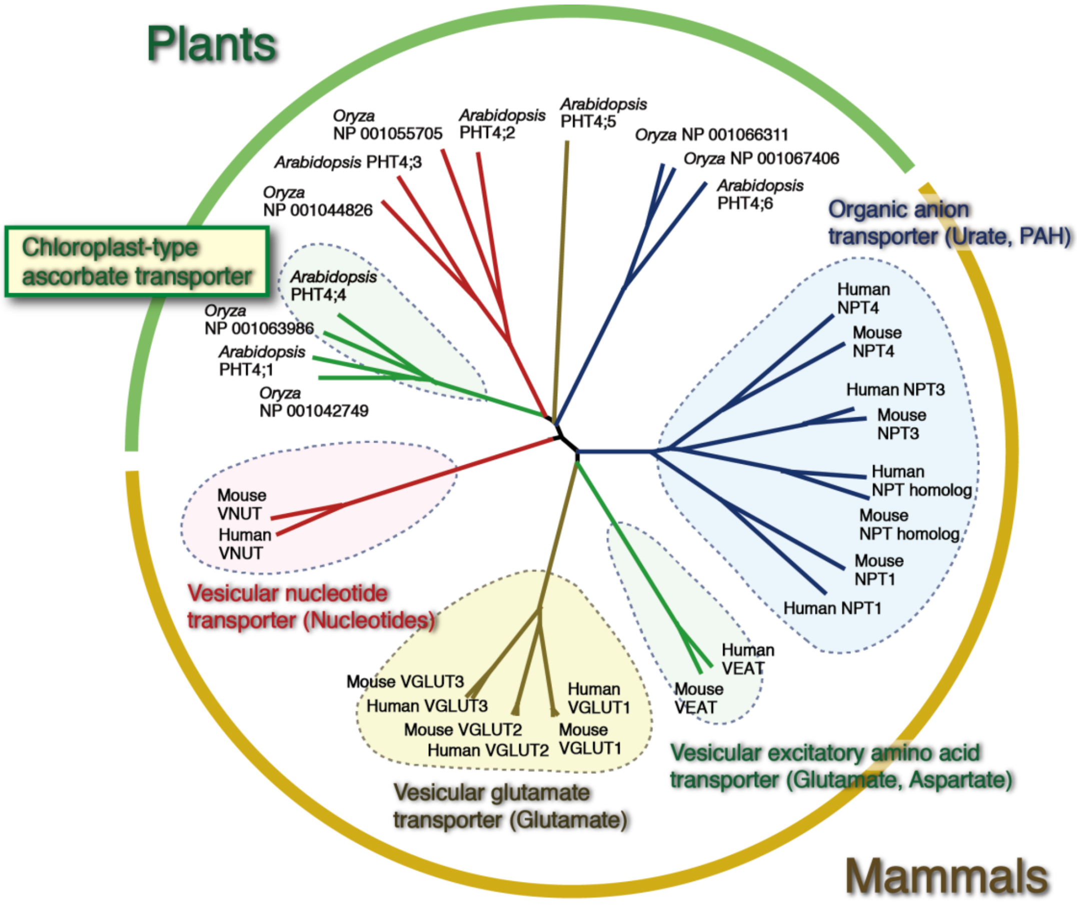

Supplementary Figure 5. Phylogenetic tree of the SLC17 transporter family in mammals and plants. SLC17 transporter family orthologues are distributed in mammals and the plant kingdom, and have evolved differently in each lineage. Phylogenetic tree showing selected genes from human, mouse, *Arabidopsis* and *Oryza*.

```
AtPHT4;4        -MALGGLISNRNFGSFIGS----GNGCQRLGKSGAEVSKLFPNALLCRNHQPLQASLHHE 55
AtPHT4;1        -MNARALLCSSNIHSLYTS----NRPPEKTSSS-----------RSLRNLKPSPKSLRVW 44
AtPHT4;2        ----MATVGSLKPLHHSSCSSSFPRNPIVNRKALLG-------FVFDSARKNQIR----- 44
AtPHT4;3        MCYSLSIQSSIDFHNRNALKIHGDRAILTSNLPTLR-------RIPFLPERDRRRKLVLC 53
AtPHT4;5        ----MARLTLRPHNHFFSSPIYAHKQP----------------FLSVYTIFPHHHQNPLI 40
AtPHT4;6        ------------------------------------------------------------

AtPHT4;4        SGHMRRSFGCFLQPRMDSVIRFRNSIKINRSRAYYKSEESDITEGVVPSADGSAEAILVE 115
AtPHT4;1        IYPRNRSSVFRVLVRSSDKSESSNSYYVEGDKVSGNND------------------VVSD 86
AtPHT4;2        ----CENLRYSSESDGKRRNAAAKKRNQSPERCAAEGVLTG------------------G 82
AtPHT4;3        TGRVVNSLKFTGNTSVDLCGIPRHRLRVSCSDARRTPEETA------------------A 95
AtPHT4;5        KSRVKCSASGTERVRESKKLPPKDPIEDPKPQLPIPEVLS------------------TE 82
AtPHT4;6        ------------------------------------------------------------

AtPHT4;4        GNLQNASPWWQQFPRRWVIVLLCFSSFLLCNMDRVNMSIAILPMSQEYNWSSATVGLIQS 175
AtPHT4;1        SPSSIVLPWWEEFPKRWVIVLLCFSAFLLCNMDRVNMSIAILPMSAEYGWNPATVGLIQS 146
AtPHT4;2        GGSEAIAEVRTMMPERIKVVILTACMMCLCNADRVVMSVAVVPLADKLGWSSSFLGVVQS 142
AtPHT4;3        ELTAQPNFSEFITSERVKVVAMLALALALCNADRVVMSVAIVPLSLSRGWSKSFSGIVQS 155
AtPHT4;5        TGFEQNWPPWKNIPQRYKLIGATSLAFVICNMDKVNLSIAIIPMSHQFGWSSSVAGLVQS 142
AtPHT4;6        -------MKLSNIPQRYVIVFLTFLSTCVCYIERVGFSIAYTVAADAAGINQSSKGTILS 53

AtPHT4;4        SFFWGYLLTQILGGIWADKFGGKVVLGFGVVWWSFATIMTPIAARLGLPFLLVVRAFMGI 235
AtPHT4;1        SFFWGYLLTQIAGGIWADTVGGKRVLGFGVIWWSIATILTPVAAKLGLPYLLVVRAFMGV 206
AtPHT4;2        SFLWGYIFSSVIGGALVDRYGGKRVLAWGVALWSLATLLTPWAAAHSTLALLCVRAFFGL 202
AtPHT4;3        SFLWGYLISPIAGGTLVDRYGGKVVMAWGVALWSLATFLTPWAADSSLWALLAARAMVGV 215
AtPHT4;5        SFFWGYALSQLPGGWLSKIFGGRKVLEIGVFTWSFATALVPLLAGF-MPGLIFSRILVGI 201
AtPHT4;6        TFFVGYACSQVPGGWAAQKIGGRKVLLLSFVLWSSTCFLVPLDPNR-VGLLVVARLLVGV 112

AtPHT4;4        GEGVAMPAMNNMLSKWIPVSERSRSLALVYSGMYLGSVTGLAFSPMLITKFGWPSVFYSF 295
AtPHT4;1        GEGVAMPAMNNILSKWVPVQERSRSLALVYSGMYLGSVTGLAFSPFLIHQFGWPSVFYSF 266
AtPHT4;2        AEGVAMPSMTTLLSRWFPMDERASAVGISMAGFHMGNVVGLLLTPLMLSSIGISGPFILF 262
AtPHT4;3        AEGVALPCMNNMVARWFPPTERSRAVGIAMAGFQLGNVVGLMLSPILMSQGGIYGPFVIF 275
AtPHT4;5        GEGVSPSAATDLIARTIPVKERSRAVGFVFGGLSLGSVMGLLLAPPIIETFNWESVFYLF 261
AtPHT4;6        AQGFIFPSIHTVLAQWVPPHERSRLVSITTSGMYLGAALGMWLLPALVELRGPESVFLAE 172

AtPHT4;4        GSLGSIWFLLWLKFAYSSPKDDP--DLSEEEKKVILGGSKPR-----EPVTVIPWKLILS 348
AtPHT4;1        GSLGTVWLTLWLTKAESSPLEDP--TLLPEERKLIADNCASK-----EPVKSIPWRLILS 319
AtPHT4;2        ASLGLLWVSTWSSGVTNNPQDSPFITRSELRLIQAGKPVQPS---TISPKPNPSLRLLLS 319
AtPHT4;3        GLSGFLWLLVWLSATSSAPDRHPQITKSELEYIKQKKQISTMENKRISTSGIPPFGRLLS 335
AtPHT4;5        GLLGVGW-FVGFQFLNEEEVSYKGNEISTSHKSENATKEELG-----SSLKEIPWKSFFQ 315
AtPHT4;6        ALAGVIWSLLWIRYATDPPRSEHPKAAAAGFGGALLPTNVNH-----HKVTHIPWKKIML 227

AtPHT4;4        KPPVWALIISHFCHNWGTFILLTWMPTYYNQVLKFNLTESGLLCVLPWLTMAVFANIGGW 408
AtPHT4;1        KPPVWALISCHFCHNWGTFILLTWMPTYYHQVLKFNLMESGLLSVFPWMTMAISANAGGW 379
AtPHT4;2        KLPTWAIIFANVTNNWGYFVLLSWMPVYFQTVFNVNLKQAAWFSALPWATMAISGYYAGA 379
AtPHT4;3        KMPTWAVIVANSMHSWGFFVILSWMPIYFNSVYHVNLKQAAWFSAVPWSMMAFTGYIAGF 395
AtPHT4;5        SPAVWAMIYTHFCGSWGHYTCLSWLPTYFSEALSLNLTEAAWVSILPPLASIVVTSLASQ 375
AtPHT4;6        SLPVWAIVVNNFTFHYALYVLMNWLPTYFELGLQISLQGMDSSKMVPYLNMFVFSIVGGF 287

AtPHT4;4        IADTLVSR-GLSITNVRKIMQSIGFLGPAFFLSQLSHVK--TPAMAVLCMACSQGSDAFS 465
AtPHT4;1        IADTLVSR-GFSVTNVRKIMQTIGFLGPAFFLTQLKHID--SPTMAVLCMACSQGTDAFS 436
AtPHT4;2        ASDFLIRT-GHSVTSVRKIMQSIGFMGPGLSLLCLNFAK--SPSCAAVFMTIALSLSSFS 436
AtPHT4;3        WSDLLIRR-GTSITLTRKIMQSIGFIGPGIALIGLTTAK--QPLVASAWLSLAVGLKSFS 452
AtPHT4;5        FADYLITN-GVDTTTVRKICQTIAFVAPAICMTLSSVDIGLPPWEIVGILTAGLALSSFA 434
AtPHT4;6        IADYLITKRILSVTRTRKFLNTVGFLIASAALMVLPMFR--TENGVILCSSVALGFLALG 345

AtPHT4;4        QSGLYSNHQDIGPRYAGVLLGLSNTAGVLAGVFGTAATGYILQR-----------GSWDD 514
AtPHT4;1        QSGLYSNHQDIAPRYSGVLLGLSNTAGVLAGVLGTAATGHILQH-----------GSWDD 485
AtPHT4;2        QAGFLLNMQDIAPQYAGFLHGISNCAGTLAAIVSTIGTGYFVQWLG-----------SFQ 485
AtPHT4;3        HLGFLINLQEIAPEYSGVLHGMCLTAGTLAAIVGTVGAGFFVELLG-----------SFQ 501
AtPHT4;5        LSGLYCTHQDISPEYASILLGITNTVGAVPGIVGVALTGFLLDS----------THSWTM 484
AtPHT4;6        RAGFAVNHMDIAPRYAGIVMGVSNTAGTLAGIIGVDLTGKLLEASKLVYSDLSHPESWRV 405

AtPHT4;4        VFKVA-VALYLIGTLVWNLFATGEKILD----- 541
AtPHT4;1        VFTIS-VGLYLVGTVIWNLFSTGEKIID----- 512
AtPHT4;2        AFLTVTAFLYFATTVFWLLFATGERVF------ 512
AtPHT4;3        GFILLTAILYLLSALFYNIYATGERVDFDTTA- 533
AtPHT4;5        SLFVPSIFFYLTGTVVWLAFASSEPQTFRKEDS 517
AtPHT4;6        VFFIPGLLCIFS-SVVFLLFSTGERIFD----- 432
```

Supplementary Figure 6. Amino acid sequence comparison of members of the AtPHT4 family.

Essential amino acid residues in the mammalian SLC17 family are indicated in red. Predicted transmembrane regions are shown in green boxes. The recognition site of AtPHT4;4 antibody is shown in a yellow box.

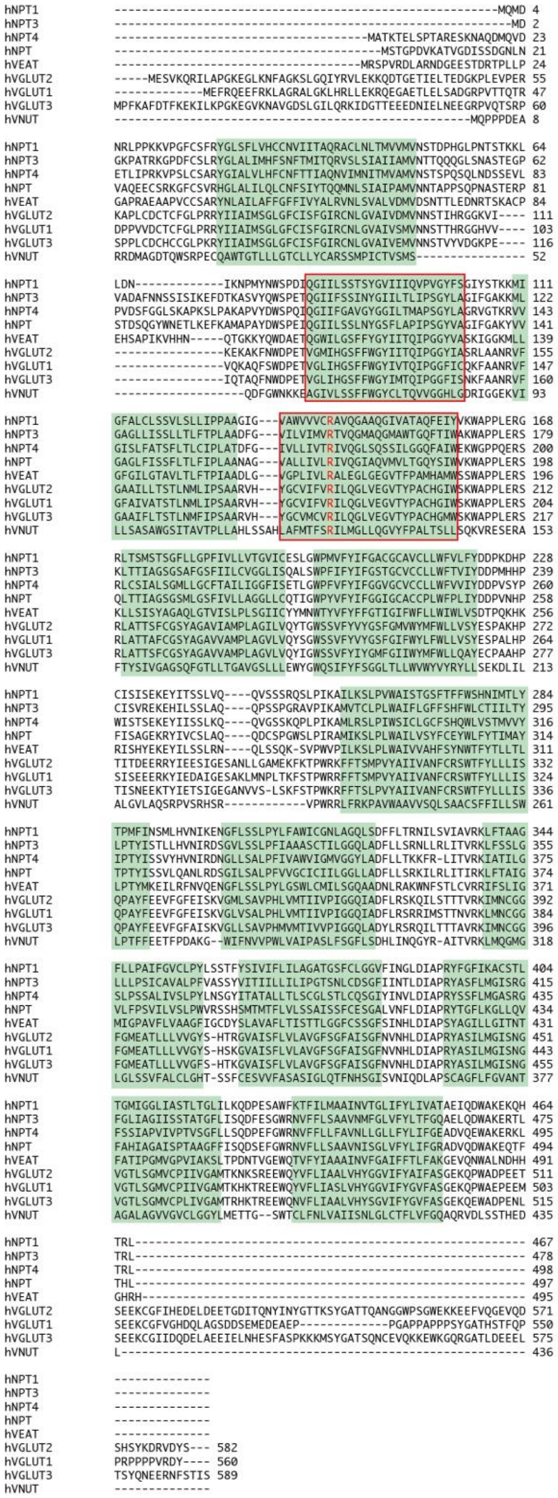

Supplementary Figure 7. Amino acid sequence comparison of members of the human SLC17 transporter family.

Essential amino acid residues and transmembrane domains in the mammalian SLC17 family are indicated in red and red boxes, respectively. Predicted transmembrane regions are shown in green boxes.

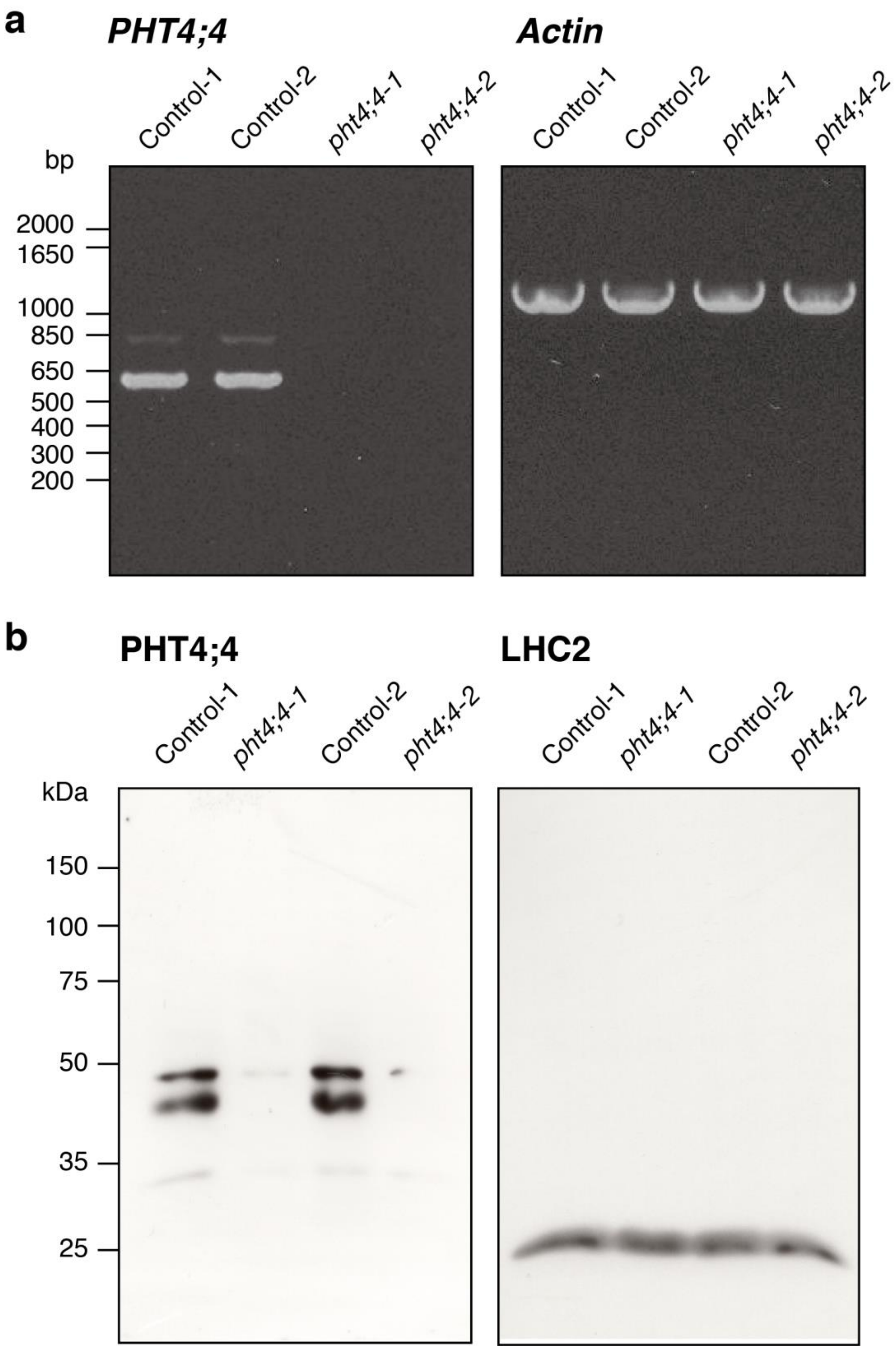

Supplementary Figure 8. Full-size images of Figure 4a (a) and 4b (b).
